# Effect of Cognitive Reappraisal on Archery Performance of Elite Athletes: The Mediating Effects of Sport-Confidence and Attention

**DOI:** 10.3389/fpsyg.2022.860817

**Published:** 2022-04-21

**Authors:** Dongling Wang, Ti Hu, Rui Luo, Qiqi Shen, Yuan Wang, Xiujuan Li, Jiang Qiao, Lina Zhu, Lei Cui, Hengchan Yin

**Affiliations:** ^1^College of P.E. and Sports, Beijing Normal University, Beijing, China; ^2^Mental Health Education and Counseling Center, China University of Labor Relations, Beijing, China; ^3^State-Owned Assets Management Office, Beijing Normal University, Beijing, China; ^4^P.E. Department, Renmin University of China, Beijing, China; ^5^BaoLong Foreign Language School, Shenzhen, China; ^6^College of Physical Education, Yangzhou University, Yangzhou, China

**Keywords:** archery, performance, sport-confidence, attention, cognitive reappraisal, PLS-SEM

## Abstract

Through empirical studies or laboratory tests, previous studies have shown that sport-confidence, attention, and emotion regulation are key factors in archery performance. The present study aims to further identify the effects and pathways of sport-confidence, attention, and cognitive reappraisal (a specific emotion regulation strategy) on real-world archery performance by constructing a hypothesized model to provide a basis for scientific training of athletes to improve sport performance. A survey design was utilized on a sample of 61 athletes (12 international-level athletes, 30 national-level athletes, and 19 first-class athletes) from the Chinese National Archery Team to test the model. The measurement and hypothesized models were tested using partial least squares structural equation modeling (PLS-SEM). The results indicate that the model fit well and explained 33.6% of the variance in archery performance. Sport-confidence (total effects = 0.574, *p* < 0.001) and attention (total effects = 0.344, *p* = 0.009) were important predictive indicators of archery performance, while the relationship between cognitive reappraisal and archery performance showed considerable complexity (direct effects = −0.268, *p* = 0.020; total effects = −0.007, *p* = 0.964). We conclude that the development of sport-confidence and attention of archery athletes should be strengthened, but athletes who use cognitive reappraisal in archery competition should be mindful of its potential appropriation of cognitive resources and should be directed to improve sport-confidence or develop a positive orientation to arouse excitement.

## Introduction

Archery performance is affected by athletes’ skills (mental, fitness, etc.), among which skills and mental abilities are key factors. The “two-point-one-line” aiming method of archery requires athletes to pursue the “stability” or “consistency” of movement skills (Stuart and Atha, 1990; Soylu et al., 2006; Sarro et al., 2021). The repeatability and consistency of skills are strongly correlated with mental ability. In addition, the current Olympic archery rules, such as the single elimination with two out of three games in three arrows per set and a single arrow shoot-off when there is a tie, greatly increase the uncertainty of the competition, make the competition extremely uncertain, and have placed higher demands on the mental ability of the athletes (Wang and Liu, 2013).

Studies have shown that archers with extensive sports experience or competition experience perform better in terms of lack of worries, confidence, and concentration (Bebetsos, 2015), or attention networks (Lu et al., 2021), and higher archery shooting scores or smaller arrow dispersion are predicted by mental abilities, such as anxiety (Zhao et al., 2013; Taha et al., 2018), confidence (Zhao et al., 2013; Taha et al., 2018; Salleh et al., 2020), concentration (Taha et al., 2018), and emotion regulation (Zhao et al., 2013; Kim and Oh, 2015). By comparing 13 mental factors, Yang et al. (2008) found that the indicators of elite archery athlete selection include the distribution of attention, kinesthetic perception, and anxiety/confidence. Similarly, by comparing 10 mental factors, Kim et al. (2015) combined the Delphi survey and hierarchical analysis to verify that confidence, concentration, and emotion control are the three most important factors affecting archery performance.

However, most previous studies describe archery performance in the form of subjective evaluations or based on tests under laboratory conditions, lacking validation with real-world archery performance; moreover, the studies (Carson and Collins, 2016; Stanger et al., 2018) have implied that there might be interactions among confidence, attention, and emotion regulation strategy, suggesting that we could investigate the relationship between them in an integrated and linked perspective. Thus, this study aims to construct a theoretical model that investigates the relationship between sport-confidence, attention, emotion regulation, and sports performance.

Based on the above statements, this study will make research contributions in the following aspects: (1) verifying the effects of sport-confidence, attention, and cognitive reappraisal on actual archery performance; and (2) exploring the path of the interaction between the above psychological factors.

## Literature Review

### Sport-Confidence and Archery Performance

The multidimensional theory of anxiety proposes that cognitive anxiety shares a negative linear relationship with performance, and confidence shares a positive linear relationship with performance (Martens et al., 1990). Research on competitive state anxiety is based on the premise that cognitive anxiety has a negative linear relationship with performance and that confidence has a linear positive relationship with performance and is considered to be a protective factor against cognitive anxiety (Weinberg and Gould, 2014). When facing stress, athletes with higher confidence are better able to interpret anxiety as a positive emotion and promote sport performance (Martens et al., 1990; Woodman and Hardy, 2003; Hays et al., 2009). Similarly, confidence has a significant effect on sport performance in archery (Zhao et al., 2013; Bebetsos, 2015), even though it affects performance to the greatest extent on different factors among skills and mental and physical fitness (Yang et al., 2008; Kim et al., 2015). In addition, several studies have examined the effects of trait anxiety (Kaur and Shenoy, 2019) and state anxiety (Lim, 2016; Kaur and Shenoy, 2019) on archery performance, but it is not clear whether there is a difference in the effect of trait sport-confidence and state sport-confidence [concepts belonging to a sport-specific model of self-confidence (Vealey, 1986)] on archery performance. In summary, we propose the following hypothesis:

*H1*: *Sport-confidence, including trait sport-confidence and state sport-confidence, positively predicts archery performance*.

### Attention and Archery Performance

Attention is described as the sustained focus of cognitive resources on information while filtering or ignoring extraneous information. It is a vital prerequisite of successful performance in sports (Abernethy et al., 2007 Moran, 2012). Athletes with a higher attention ability are better able to achieve desirable performance in competition (Moran, 1996; Love et al., 2018). Likewise, researchers have concurred that attention, especially concentration, is critical for archery athletes (Lee, 2009; Zhao et al., 2013; Bebetsos, 2015; Taha et al., 2018). Gonzalez et al. (2017) pointed out that longer quiet eye durations (QED) are required for archers to aim accurately at the bullseye, and they noted that attention control plays an important role in determining QED performance. A study focusing on athlete selection indicated that attention allocation is an important indicator for selecting elite archers (Yang et al., 2008). Lu et al. (2021) also found that national team archers performed better than provincial team archers in terms of attentional network efficiency. During the aiming process, archers need to rely on their own proprioception and environmental information (e.g., wind direction) to adjust their posture or aiming skills (Lu et al., 2021), which requires strong attention allocation and a certain breadth of attention to cope with and process this information. At the same time, athletes seem to invoke conflict control to shift attention from distracting factors (e.g., focusing on the performance of other athletes or caring about ranking) to valid information (e.g., paying attention to coordinated body movements). Thus, following the facts above, the different aspects of attention, that is, shifting, breadth, and allocation, may all influence the archery performance. In summary, hypothesis 2 is proposed:

*H2*: *Shifting, breadth, stability, and allocation of attention positively predicts archery performance*.

### Emotion Regulation and Archery Performance

In sports (e.g., archery) that require movement of fine control (Eysenck and Calvo, 1992; Mellalieu et al., 2004, 2009) or a high level of concentration (Kubiak et al., 2019), anxiety could provoke a decline in sport performance. Emotion regulation is often used by individuals as a means of reducing anxiety (Cisler et al., 2010). Admittedly, emotional control or emotion regulation is an essential mental skill for archery athletes (Yang et al., 2008; Zhao et al., 2013; Kim et al., 2015). However, previous research has progressed mostly to emphasize the importance of emotion regulation, and there has been less exploration of specific means of emotion regulation applicable to archery athletes. Although there are a considerable number of ways to regulate emotions, one prominent and adaptive approach to control emotions is through cognitive reappraisal (Gross and John, 2003; Gross, 2010; Uphill et al., 2012). Studies have found that positive cognitive reappraisal is positively associated with motor performance in table tennis, whereas expression suppression is not (Kubiak et al., 2019), and it enhances archery athletes’ feelings of positive emotions and improves performance on experimental tasks (Kim and Oh, 2015). In addition, similar to cognitive reappraisal, arousal reappraisal prevents golfers from “choking” and to some extent ensures performance in putting tasks (Balk et al., 2013). In conclusion, we chose cognitive reappraisal as a specific strategy for emotion regulation and proposed the following hypothesis:

*H3*: *Cognitive reappraisal positively predicts archery performance*.

### Sport-Confidence and Attention

According to attentional control theory, anxiety impairs the goal-directed attention system and reduces individuals’ performance on attention control by inhibiting attentional transfer processes (Eysenck et al., 2007; Eysenck and Derakshan, 2011). Among archery athletes, it has also been shown that concentration increases when competitive anxiety decreases (Indahwati and Ristanto, 2016). Multidimensional anxiety theory states that sport-confidence is an important mental quality for individuals to avoid cognitive anxiety (Martens et al., 1990). Athletes with high levels of confidence improve their attentional control and motor performance by investing more mental effort, even when they are anxious (Eysenck and Calvo, 1992). As evidence, a recent questionnaire-based study showed that high levels of attentional control are related to lower levels of competitive state anxiety and higher levels of self-confidence, and competitive state anxiety and self-confidence are significant predictors of attentional control (Tomé-Lourido et al., 2019). As a result, sport-confidence may ensure attention control by resisting anxiety and maintaining confidence. Studies on shooting athletes found that confidence had a relatively greater effect on concentration among the mental skills of target setting, representational and anxiety control (Mun, 2011), suggesting to us that confidence contributes to shifting, breadth, and allocation of attention. In summary, it is proposed the following hypotheses:

*H4*: *Sport-confidence positively predicts attention in archers*.

*H4a*: *Attention mediates the relationship between sport-confidence and archery performance*.

### Sport-Confidence and Cognitive Reappraisal

Research on the relationship between confidence and cognitive reappraisal in sports is relatively scarce. We only know that cognitive reappraisal can enhance subjects’ confidence in memory tasks (Richards and Gross, 2000). Studies on arousal reappraisal have shown that the reappraisal group displayed higher self-confidence than the control group when completing a pressurized dart-throwing task (Sammy et al., 2017). Along these lines, cognitive reappraisal affects emotion-related outcomes by modulating the intensity of emotional responses (Gross, 2010) and stress responses (Jamieson et al., 2013), such as reducing anxiety (Hofmann et al., 2009; Uphill et al., 2012; Efinger et al., 2019) and depression (Uphill et al., 2012) and shifting negative stress states (Jamieson et al., 2013). A study of a sport-specific model of self-confidence also states that these negative emotions are related to sport-confidence (Vealey, 1986). In addition, archery athletes’ sport-confidence is significantly and negatively correlated with competition anxiety and negative emotions (Zhao et al., 2013; Lim, 2016). Furthermore, according to the theory of self-efficacy, emotions are one of the sources of self-efficacy, and successful regulation of emotional experiences can enhance individuals’ perceptions of personal competence (Bandura, 1977a,b). As a result, cognitive reappraisal may promote sport-confidence by improving emotional experience and consequently affect sports performance. Therefore, we propose the following hypotheses:

*H5*: *Cognitive reappraisal positively predicts archers’ sport-confidence*.

*H5a*: *Sport-confidence mediates the relationship between cognitive reappraisal and archery performance*.

*H5b*: *Sport-confidence and attention have a chain mediating effect of cognitive reappraisal and attention*.

### Attention and Cognitive Reappraisal

Both attention and cognitive reappraisal are related to cognitive control (Ochsner and Gross, 2005; Buschman and Miller, 2010). Although cognitive reappraisal may be associated with lower cognitive costs (Gross and Thompson, 2007), recent research suggests that cognitive reappraisal depletes cognitive resources (Sheppes and Meiran, 2008; Keng et al., 2013). The use of cognitive reappraisal requires the invocation of cognitive processes, including working memory, task switching, and antagonistic dominance responses (Ortner et al., 2016; Gan et al., 2017), which may conflict with attentional control and attentional orientation. Under the condition of stress, cognitive reappraisal not only takes away self-control resources but also reduces the control of negative emotions (Sheppes and Meiran, 2007; McRae et al., 2012; Ortner et al., 2016). When athletes deliberately seek to be “more in control” to gain control over emotions and movement skills, it often comes at the cost of depleting more cognitive resources, as evidenced by decreased performance on reaction time tasks and increased rates of missed and false manipulation tasks (Sun et al., 2013; Ortner et al., 2016). Thus, we propose the following hypotheses:

*H6*: *Cognitive reappraisal negatively predicts attention in archers*.

*H6a*: *Cognitive reappraisal mediates the relationship between sport-confidence and attention*.

**Figure 1:**
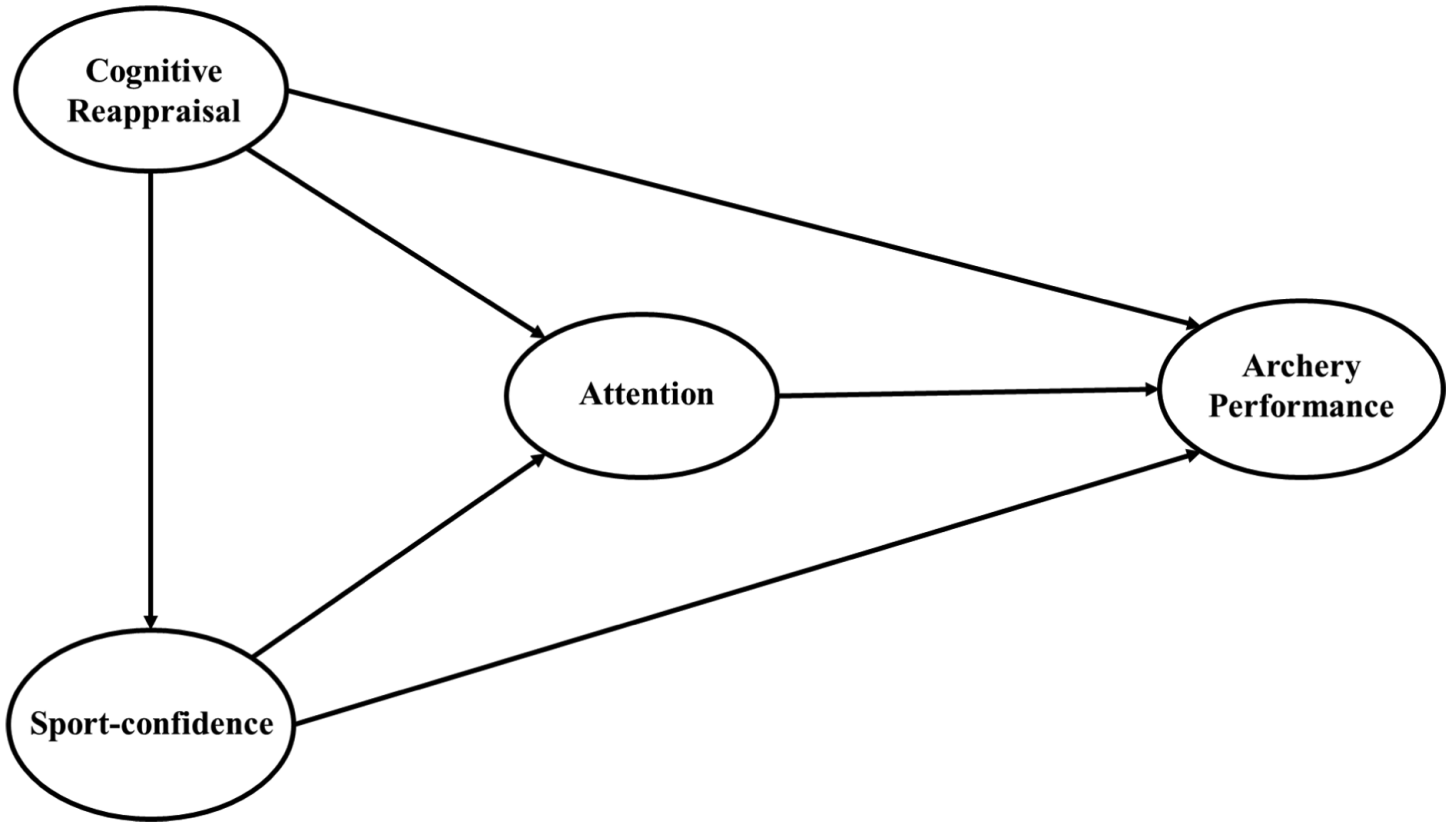
Structural model.

Combining the hypotheses above, a theoretical model of archery athletes’ sport-confidence, attention, and cognitive reappraisal influencing sport performance was obtained, as shown in Figure 1.

## Materials and Methods

### Participants

Sixty-one athletes from the Chinese National Archery Team participated in the study. Among them, 26 were males and 35 were females, 12 were international-level athletes, 30 were national-level athletes, and 19 were first-class athletes. These technical hierarchies were officially determined by the General Administration of Sport of China. International-level athletes are those who have won certain competition placement in international competitions, national-level athletes are those who have achieved corresponding competition results international, continental, or national competitions, and first-class athletes are those who have won certain competition results in national competitions (General Administration of Sport of China, 2019). Informed consent was obtained from all athletes and coaches before testing. The detailed demographic distribution is presented in Table 1.

**Table 1:** Participants’ demographics.

Gender	Technical level	*N*	Age (*M* ± SD)	Training years (*M* ± SD)
Male	International	6	23.50 ± 1.23	10.67 ± 1.86
	National	17	22.12 ± 2.83	8.88 ± 3.02
	First-class	4	16.50 ± 1.29	5.75 ± 2.06
Female	International	6	27.17 ± 2.86	13.33 ± 0.52
	National	13	20.15 ± 2.97	8.54 ± 2.47
	First-class	15	19.80 ± 2.34	7.73 ± 2.34
**Total**		61	21.39 ± 3.51	8.93 ± 2.94

*M*, *Mean; SD, standard deviation*.

### Measurements

#### Archery Performance

To avoid the bias arising from the description of sports performance by a single indicator and take into account the description of athletes’ sports performance in both long-term and short-term dimensions, we constructed an archery performance indicator that includes competition performance score (the total ranking score in the qualification and the total set score in the matchplay) and technical hierarchy score.

According to Olympic archery competition rules, athletes are required to participate in the qualification phase and the matchplay phase successively. The performance in the qualification phase is usually reflected by the total ranking score (total score of shooting 72 arrows), which could reflect the stability of the athletes’ performance; the performance in the matchplay phase is reflected by the total set score (cumulative score of each match), which can reflect the confrontational ability of the athletes. In addition, referring to the scoring method of Arribas-Galarraga et al. (2017) for the competition places achieved by athletes in different levels of competition, we devised a method for converting technical level into technical level score, that is, the second-class level, the first-class level, the national level, and the international level were assigned 1, 2, 3, and 4, respectively. Thus, the technical level score of the second-class athletes was 1 (1), that of the first-class athletes was 3 (1 + 2), that of the national-level athletes was 6 (1 + 2 + 3), and that of the international-level athletes was 10 (1 + 2 + 3 + 4).

#### Attention

The paper–pencil version of the Elite Athlete Attention Test (EAAT; Yin et al., 2006) was selected to measure the attentional allocation, breadth, and shifting of athletes. The EAAT, based on theory related to trials of attention, consists of the Graphic Discrimination Test (subjects were asked to find 2 target shapes among 16 similar shapes and mark them, the total number of shapes being 15 × 20 = 300), the Pick 4 Circles Test (subjects were asked to mark the squares with four small circles among the squares with different numbers of small circles, the total number of squares being 26 × 25 = 650), and the Addition and Subtraction Test (subjects were asked to alternate between “addition” and “subtraction” and to write the results in the middle of the two numbers, the total number of results being 22 × 12 = 264), which measure attentional allocation, breadth, and shifting, respectively. The validity of the EAAT has been proven in national team athlete populations in tennis, diving, short track speed skating, basketball, free-skiing aerials, and sailing, by comparing the attention ability with those of the self-assessment and the coaches’ assessment (Yin et al., 2006).

#### Sport-Confidence

The State Sport-Confidence Inventory (Chinese Version; SSCI-CV; Fung et al., 2001) was chosen to measure the state sport-confidence in this study. The SSCI-CV revised from the State Sport-Confidence Inventory (SSCI; Vealey, 1986), which has been a widely used measure of state sport-confidence and has shown good reliability and validity. It consists of 13 items (e.g., “compare your confidence in your ability to perform under pressure to the most confident athlete you know”) and each scored on a 9-point Likert scale from 1 (low confidence) to 9 (high confidence). The internal consistency of the SSCI-CV was 0.94 in this study.

The Athlete Sport-Confidence Inventory (ASCI; Shen, 2004) was chosen to measure the trait sport-confidence in this study. The ASCI is an instrument for measuring trait sport-confidence based on self-efficacy theory (Shen, 2004), sport-specific model of self-confidence (Vealey, 1986), and the Chinese elite athlete self-confidence model (Anmin et al., 1999). Compared to the Trait Sport-Confidence Inventory (TSCI; Vealey, 1986), the ASCI incorporates competition orientation, which can be a valid predictor of trait sport-confidence (Martin and Gill, 1991; Shen, 2004) and would more comprehensively measure and reflect athletes’ trait sport-confidence. The ASCI consists of 16 items and each scored on a 6-point Likert scale ranging from 1 (not at all) to 6 (very much so). It is designed to measure the trait task sport-confidence (six items, e.g., “Even with a lot of pressure, I was able to play well as I should”) and trait coping sport-confidence (10 items, e.g., “Whether I win or lose, I believe I can reach the goal I have in mind”). The test–retest reliability and the coefficient of were 0.78 and 0.84 for task dimension and were 0.73 and 0.90 for coping. In this study, the internal consistency of the ASCI was 0.94.

#### Cognitive Reappraisal

The Emotion Regulation Questionnaire-Chinese Revised Version (ERQ-CRV; Wang et al., 2007), a revised version of the Emotion Regulation Questionnaire (ERQ; Gross and John, 2003), was selected to measure the use of cognitive reappraisal strategies. The ERQ has been a widely used measure of emotion regulation strategies and has shown good reliability and validity. The ERQ-CRV comprises two subscales and assesses the habitual use of two emotion regulation strategies: cognitive reappraisal and expressive suppression. This study applied the Cognitive Reappraisal Scale, which consists of six items (e.g., “When I’m faced with a stressful situation, I make myself think about it in a way that helps me stay calm”), and each scored on a 7-point Likert scale ranging from 1 (strongly disagree) to 7 (strongly agree). The test–retest reliability and the coefficient of ERQ-CRV were 0.82 and 0.85 for cognitive reappraisal dimension. The internal consistency coefficient was 0.80 in this study.

### Procedure

All of the athletes took the psychological test as a group at the same time and participated in an archery competition 1 week after the test. The psychological tests were conducted 2 h after the end of training in a warm, well-ventilated, and quiet room. Before implementing the attention tests, the chief examiner explained each subtask of the EAAT one by one and confirmed that the athletes understood the content of the EAAT, and then counted down the time to start the attention tests on the athletes. All three subtasks of the EAAT were limited to 3 min (Yin et al., 2006). After completing the attention tests, the athletes filled out the ERQ-CRV, the SSCI-CV, and the ASCI. The archery competition was conducted in accordance with Olympic archery competition rules. During the competition, we collected the total ranking score in qualification phase and the total set score in the matchplay phase. In addition, the technical level data are collected from the “Athlete Technical Hierarchy Integrated Management System” before the competition.

### Statistical Analysis

In this cross-sectional study, structural equation modeling (SEM) was chosen to analyze the measurement and structural models. SEM was divided into two categories: covariance-based SEM (CB-SEM) and partial least squares-SEM (PLS-SEM; Chin, 1998). Compared to CB-SEM, PLS-SEM (1) is more suitable for addressing small sample data and does not require normally distributed data (Chin, 1998; Urbach and Ahlemann, 2010); (2) focused on predictions and theory building, rather than model verification, by maximizing the explained variance of endogenous latent variables because its power is relatively higher (Hair et al., 2011); and (3) The model constructed in this study is permeated with various theories or hypotheses and is a development of theory rather than a validation of the theory, which determines the applicability of PLS-SEM for this study. The data were analyzed in SmartPLS V.3.3.3 (Ringle et al., 2015).

We assessed the model using a two-stage analytical procedure for PLS-SEM as delineated by Hair et al. (2020): (1) We tested the measurement model to refine our measures and establish validity and reliability; and (2) We examined the structural model and tested the significance of path coefficients. The PLS algorithm was utilized to calculate the weights/loading, path coefficients, predictive accuracy (*R^2^*), effect size (*f^2^*), reliability, and validity of the model; bootstrapping (resamples = 5,000, confidence intervals method is bootstrap with corrected bias-corrected and accelerated bootstrap) was utilized to calculate the statistical significance of path coefficients; and blindfolding (omission distance = 7) was utilized to calculate Stone-Geisser’s *Q*^2^, which indicates predictive relevance (Hair et al., 2017). The remaining calculation parameters were the software’s default values. To comply with the rule of the “minimum sample size should be equal to the larger 10 times the largest number of formative indicators used to measure one construct sample” in the PLS-SEM algorithm (Marcoulides and Saunders, 2006), we packaged the SC-state and SC-trait in the form of summation of each item’s score.

## Results

### Measurement Model

**Figure 2:**
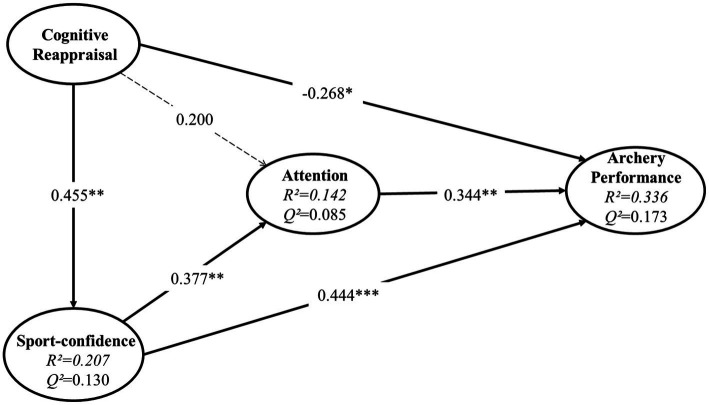
Structural model and PLS-SEM results. ^*^p < 0.05, ^**^p < 0.01, ^***^p < 0.001.

Items for which the indicator loading is less than 0.6 should not be included in the subsequent analysis (Sarstedt et al., 2017). The preliminary PLS-SEM algorithms showed that the outer loading of the first and second items of the latent variable cognitive reappraisal ranged from 0.475 and 0.586. Based on the results of both PLS algorithms and empirical analyses (the two items are correlated in connotation with other items, and removing them does not affect the content validity), we deleted the two items above. Meanwhile, the relationship between cognitive reappraisal and attention (H6) was removed from the original hypothesized model due to a non-significant path coefficient [*β* = 0.200, 95% CI ∈ (−0.069, 0.449)]. The following section will develop the analysis with the revised model (Figure 2).

To measure reliability, indicator loadings higher than 0.7, composite reliability (*CR*) ranging from 0.8 to 0.95, and Cronbach’s alpha (*CA*) higher than 0.7 have been used as common criteria to assess indicator reliability and internal consistency reliability (Sarstedt et al., 2017). However, some researchers criticized *CA* as an indicator to assess the reliability of the measurement model in PLS-SEM, in that the calculation of *CA* is based on the assumption that “all items have tau-equivalence reliability,” which may underestimate the true reliability (Hair et al., 2012; Peterson and Kim, 2013). As shown in Table 2, indicator loadings (0.708–0.912) of each item, *CR* values (0.826–0.897) of each latent variable, and *CA* values (0.771–0.807) of the latent variables above cognitive reappraisal, sport-confidence, and attention comply with the requirements of the test standard. Although the *CA* value for the latent variable archery performance was 0.696, we considered the *CA* for archery performance to be acceptable due to the inherent large uncertainty in archery performance and the general tendency of *CA* to underestimate the reliability. In all, the measurement model was considered to have acceptable reliability in this study.

**Table 2:** Reliability tests and validity tests of the measurement model.

Constructs	Loadings range	*CA*	*CR*	*AVE*
Archery Performance	0.756–0.835^***^	0.696	0.826	0.614
Sport-confidence	0.893–0.912^***^	0.772	0.897	0.814
Attention	0.793–0.874^***^	0.771	0.866	0.684
Cognitive Reappraisal	0.708–0.851^***^	0.807	0.872	0.631

*CA*, *Cronbach’s alpha; *CR*, composite reliability; *AVE*, average variance extracted*.

*** *p < 0.001*.

The validity tests of measurement models focus on convergent validity and discriminant validity tests (Hair et al., 2020). Convergent validity, which reflects the extent to which different measures of the same construct converge or strongly correlate with one another (Taherdoost, 2016), was assessed through the average variance extracted (*AVE*). All *AVEs* (Table 2) in this study exceeded the recommended threshold of 0.5 (Hair et al., 2020), indicating that the measurement variables can effectively explain the latent variables and that the measurement models were both good in convergent validity. The discriminant validity assessment ensures that a reflective construct has the strongest relationships with its own indicators (Hair et al., 2020). Traditionally, the Fornell–Larcker criterion (Fornell and Larcker, 1981) discriminated whether $\sqrt{AVE_{i}}>\max\;\left|r_{ij}\right|\left(\forall i\neq j\right)$ was used, but Henseler et al. (2015) pointed out that the PLS algorithm would overestimate indicator loadings and underestimate correlation coefficients among the constructs, and they suggested that a heterotrait–monotrait ratio of correlations (HTMT) lower than 0.85 should be used as a criterion of discriminant validity assessment. Tables 3 and 4 present the results of these criteria. Both the Fornell–Larcker criterion and HTMT test were approved. Therefore, we demonstrated satisfactory validity of our measurement model.

**Table 3:** Fornell–Larcker criterion analyses.

Constructs	1	2	3	4
1. Archery Performance	**0.784**			
2. Attention	0.426	**0.827**		
3. Cognitive Reappraisal	0.044	0.320	**0.794**	
4. Sport-confidence	0.452	0.377	0.455	**0.902**

*The numbers in bold on the diagonal in the matrix of correlation are the square roots of the* *AVE*.

**Table 4:** Heterotrait–monotrait (HTMT) analysis.

Constructs	1	2	3	4
1. Archery Performance				
2. Attention	0.545			
3. Cognitive Reappraisal	0.165	0.374		
4. Sport-confidence	0.583	0.476	0.545	

### Structural Model

Assessing the structural model, we checked for multicollinearity issues by examining the variance inflation factor (VIF) values first. No multicollinearity issues were found, as all VIF values, ranging from 1.272 to 2.370, were below the threshold value of three (Hair et al., 2020). As shown in Figure 2, the endogenous variables’ *R*^2^ value (range from 0.142 to 0.336) and *Q*^2^ value (range from 0.085 to 0.173) also indicated good predictive accuracy and predictive relevance, where *R*^2^ equals 0.67, 0.33, and 0.19 for large, medium, and small predictive power, respectively, and *Q*^2^ values greater than zero are meaningful, whereas values below zero indicate a lack of predictive relevance.

### Direct Path Analysis

To assess the direct effect of the path, path coefficients’ significance (*p*) and effect size (*f  ^2^*) were used, in which *f  ^2^* values above 0.02 and up to 0.15 are considered small effects, values of 0.15 and up to 0.35 are considered medium effects, and values 0.35 and above are considered large effects (Cohen, 1988). The relationships between sport-confidence and archery performance (H1; *β* = 0.444, *p* < 0.001), sport-confidence and attention (H4; *β* = 0.377, *p* < 0.001), and cognitive reappraisal and sport-confidence (H5; *β* = 0.455, *p* < 0.001) were found to have a medium effect size. Small effects were observed for the relationships between attention and archery performance (H2; *β* = 0.344, *p* < 0.001) and between cognitive reappraisal and archery performance (H3; *β* = −0.268, *p* < 0.001; see Table 5). Overall, these results support H1, H2, H4, and H5. Beyond our anticipation, the results do not support H3, but a significant negative relationship between cognitive reappraisal and archery performance was found. In addition, no significant relationships were found between cognitive reappraisal and attention (H6), which had been clarified in the model revision process.

**Table 5:** Path coefficients of the structural model and significance testing results.

Path of the research model	*Beta*	*95% CI*		*f* *^2^*	Hypothesis decision
		2.5%	97.5%		
1. Sport-confidence → Archery Performance	0.444^***^	0.207	0.684	0.218	H1 is supported
2. Attention → Archery Performance	0.344^**^	0.068	0.581	0.148	H2 is supported
3. Cognitive Reappraisal → Archery Performance	−0.268^*^	−0.486	−0.036	0.083	H3 is not supported
4. Sport-confidence →Attention	0.377^**^	0.148	0.588	0.166	H4 is supported
5. Cognitive Reappraisal → Sport-confidence	0.455^**^	0.044	0.684	0.261	H5 is supported
6. Cognitive Reappraisal → Attention	0.200	−0.069	0.449	–	H6 is not supported
7. Sport-confidence → Attention → Archery Performance	0.130^*^	0.021	0.257	–	H4a is supported
8. Cognitive Reappraisal → Sport-confidence → Archery Performance	0.202^*^	0.011	0.419	–	H5a is supported
9. Cognitive Reappraisal → Sport-confidence → Attention → Archery Performance	0.059	−0.004	0.145	–	H5b is not supported
10. Cognitive Reappraisal → Sport-confidence → Attention	0.172^*^	0.009	0.354	–	H6a is supported

* *p* *< 0.05;*

** *p* *< 0.01;*

*** *p < 0.001*.

### Mediation Analysis

The mediation analysis procedure recommended by Hair et al. (2017) was used, in which, if the coefficient of the path including the mediator variable is significant, then we need to determine the significance of the direct effect. Partial mediation is found when the direct effect is significant, whereas support for full mediation is demonstrated when the direct effect is not significant. Among the results of the mediating effects test (Table 5), we found that attention acted as a partial mediator of the relationships between sport-confidence and archery performance (H4a, *β* = 0.130, *p* < 0.05). Sport-confidence acted as a suppressing mediator between cognitive reappraisal and archery performance (H5a, *β* = 0.202, *p* < 0.05) due to the opposite sign of the coefficient of the indirect effect and the coefficient of the direct effect (*β* = −0.268, *p* < 0.05). Moreover, a mediating effect of sport-confidence was found in the path between cognitive reappraisal and attention (H6a, *β* = 0.172, *p* < 0.05). Certainly, sport-confidence and attention have no chain mediating effect between cognitive reappraisal and attention (H5b, *β* = 0.059, *p* > 0.05). Overall, the results of the indirect relationships provided support for H4a, H5a, and H6a, and did not support H5b.

### Total Effect Analysis

To compare the magnitude of different independent constructs affecting the dependent constructs, we collated the total effects of sport-confidence, attention, and cognitive reappraisal to influence archery performance by summing the direct and indirect effects. As shown in Table 6, the ranking of the total effects of the different constructs on archery performance was, in descending order, sport-confidence [0.574, 95% CI ∈ (0.342, 0.796)], attention [0.344, 95% CI ∈ (0.068, 0.581)], and cognitive reappraisal [−0.007, 95% CI ∈ (−0.354, 0.271)].

**Table 6:** Direct effects and indirect effects of independent constructs among Archery performance.

Constructs	Sport-confidence	Attention		Archery Performance		
	Direct	Direct	Indirect	Direct	Indirect	Total
1. Sport-confidence	–	0.377^**^	–	0.444^***^	0.130^*^	0.574^***^
2. Attention	–	–	–	0.344^**^		0.344^**^
3. Cognitive Reappraisal	0.455^**^		0.172^*^	−0.268^*^	0.261^*^	−0.007

* *p* *< 0.05;*

** *p* *< 0.01;*

*** *p < 0.001*.

## Discussion

The main purpose of this study was to explore the pathways and effects of sport-confidence, attention, and cognitive reappraisal on real-world archery performance from a connected perspective. Before proving the hypothesized relationships, we verified that the measurement models had acceptable internal consistency reliability, convergent validity, and discriminant validity. In addition to demonstrating again that sport-confidence and attention are important predictive indicators of archery performance (Yang et al., 2008; Kim et al., 2015; Taha et al., 2018), we also found complexity when cognitive reappraisal affects archery performance. Furthermore, the fact that the model explained a total of 33.6% of the variance in archery performance is in line with our expectations and the conclusions of a previous study (Kim et al., 2015), considering that the factors influencing archery performance also include skill-based factors, physical fitness-based factors, and other mental factors.

Echoing the hypothesis of a positive linear relationship between sport-confidence and archery performance proposed by Martens et al. (1990) in multidimensional anxiety theory, this study demonstrated that sport-confidence has a positive effect on archery performance. Stable technical performance comes from stable mental dominance, which depends heavily on confidence (Yang et al., 2008; Kim et al., 2015). Consistent with the findings of Kim et al. (2015), we reconfirm that confidence has the greatest effect on archery performance compared to attention and emotion regulation and state the primacy of developing sport-confidence in archers. Moreover, the results support the assumption that both state and trait sport-confidence are equally important, which gives us reason to speculate that an athlete’s past success is an important prerequisite for optimal performance at high levels of competition (Rosenqvist and Skans, 2015) and suggests that the development of sport-confidence in archers can be carried out in terms of improving the degree of belief in using their abilities to accomplish their sporting tasks and the degree of belief in facing pressure and overcoming difficulties. Therefore, paying attention to improving the sport-confidence of athletes in diversified ways is a necessary part of daily training and pre- and post-competition training.

The present study found that archery performance was affected by multiple attentional abilities, including shifting, breadth, stability, and allocation of attention. Yang et al. (2008) focused on attentional stability and attentional assignment, and Lu et al. (2021) based on attention network theory, found that the ability to alert, orient, and conflict control was related to archery performance. These studies of different attentional elements all point to the importance of abilities in maintaining focus, identifying targets and distractors, and rapidly shifting the object of attention. In other words, to ensure consistent technical performance during the competition, archery athletes need to keep their focus stabilized on the target and bow sight, along with controlling their body movements, which demands attentional breadth and attentional allocation at the same time (Pei and Song-ping, 2007). In turn, there are a large number of distractions in the competition diverting the athlete’s attention, requiring the athletes to shift the direction of their attention and keep the focus on valid information or the task at hand.

In addition, attention plays a partially mediating role in the path of sport-confidence to archery performance. The findings support the hypothesis of processing efficacy theory (Eysenck and Calvo, 1992) and attentional control theory (Eysenck and Derakshan, 2011) regarding the relationship between anxiety and attention. The lack of confidence in athletes is often manifested by the inability to focus well on the process of the game but more on the opponent’s performance or the outcome of the game, which negatively affects their normal performance on a technical level (Yang et al., 2008). If the inverse relationship between confidence and anxiety holds true, it may also suggest that anxiety has a more negative effect on performance when the task is more demanding on the central executive system (Eysenck and Derakshan, 2011). In short, we can infer that individuals with higher levels of sport-confidence are more likely to maintain and control their attention on valid information and operational tasks, thus ensuring performance.

Previous studies have indicated emotion regulation (Kim and Oh, 2015) or emotion control (Yang et al., 2008; Zhao et al., 2013; Kim et al., 2015) as an important factor in archery performance. However, we found that the relationship between cognitive reappraisal and archery performance is considerably complex. The direct effect of cognitive reappraisal on archery performance was significantly less than zero, while the total effect was approximately equal to zero and non-significant. Differences in the measurement of emotion regulation and differences in contexts when using cognitive reappraisal strategies may have contributed to the different results of the studies.

We chose emotion regulation strategies as an entry point to measure athletes’ emotion regulation ability from the “method” perspective and investigated athletes’ cognitive reappraisal, which is a relatively efficient emotion regulation strategy. Most previous studies have investigated the importance of control emotions based on “outcome” orientation, e.g., “The importance of emotional control on archery performance is …” (Yang et al., 2008; Kim et al., 2015), or “I lose control of my emotions under stress,” “My emotions prevent me from performing at my best in competition” (Zhao et al., 2013). From this, we can realize that although emotion regulation is crucial for athletes to achieve good performance, the specific methods used in regulating emotions still need to be treated differently.

Context is an important factor to consider in determining the usefulness of a particular emotion regulation strategy (Gross and John, 2003; Kucharski et al., 2018). The presence of contextual differences between the two may also account for the differential effects of cognitive reappraisal. Some scholars (Sheppes and Meiran, 2007; Ortner et al., 2016) have pointed out that the effectiveness of using cognitive reappraisal to improve negative emotional experiences in emotionally stressed conditions is lower than that of using it in non-stressed conditions. In the present study, sport performance was collected under real-world competition, while other similar studies (Wagstaff, 2014; Kim and Oh, 2015; Lane et al., 2016) measured performance under laboratory settings. In competitive situations where individuals are constantly faced with new stimuli of emotional arousal, the use of cognitive reappraisal strategies not only fails to effectively regulate emotions but also eventually leads to enhanced emotional instability and adversely affects performance as the frequency of use increases and the effectiveness of use decreases (Sheppes and Meiran, 2007; Ortner et al., 2016). Athletes tended to use more complex strategies (e.g., cognitive reappraisal) following a disappointing game or personal performance (Kucharski et al., 2018). However, increased cognitive costs are associated with the use of cognitive reappraisal strategies in a higher intensity stimuli context (Sheppes and Meiran, 2008; Ortner et al., 2016), or the cognitive reappraisal and attention spent on the task would compete for limited cognitive resources. From this, it can be inferred that archery performance is negatively influenced by cognitive reappraisal due to deviations from conventional behavioral patterns or the occurrence of additional expenditure of cognitive resources by athletes. This is in line with the ego-depletion theory (Baumeister et al., 1998).

In addition, the suppressing effect of sport-confidence between cognitive reappraisal and archery performance suggests that if athletes direct their cognitive reappraisal toward boosting sport-confidence, the use of the strategy might facilitate archery performance. This is in line with the importance to archers of positive thinking (Kim et al., 2015). This might be interpreted as individuals reappraising their anxious arousal as excitement and consequently increasing their confidence and improving their performance (Stanger et al., 2018). Through self-statements, such as “I am excited” rather than “trying to relax or stay calm,” cognitive reappraisal promotes a more positive way of thinking and elicits a stronger feeling of excitement, which in turn indirectly affects sports performance (Brooks, 2014). In summary, athletes, at least archers, should understand that using cognitive reappraisal in the field tends to reduce competitive performance by taking up cognitive resources and distracting attention, but when it is applied in a way that enhances confidence (e.g., “the current situation is a challenge for me, and I believe I can handle it”), it may promote competitive performance.

### Limitations and Future Research

The primary limitations found in this study concerned the cross-sectional nature of data collection. Cross-sectional designs limit the causality in the hypothesized relationships by examining only the relations among the variables; hence, we cannot assert that the antecedents are causally related to desired outcomes. In addition, given the difficulty of reaching 61 national archery athletes at the same time and place, we could not guarantee collecting data periodically or conducting a complete experimental study. As a result, future research could further test the model using a longitudinal design or quasi-experiment to make the findings more convincing.

The model constructed in this study involved three psychological predictive constructs and explained 33.6% of the variance in archery performance. However, cognitive reappraisal may not be a one-size-fits-all approach to regulate emotion in archery competition. We not only encourage additional investigations to further verify the psychometric properties of the measures used in this study, but also suggest the inclusion of other constructs of emotion regulation in the model, such as acceptance. Acceptance encourages individuals to become aware of and accept their feelings, rather than trying to actively change them, and it not only reduces negative perceptions of unpleasant emotional and physiological states and maintains or increases physiological arousal (Desbordes et al., 2015; Feldman et al., 2016), but more importantly, it occupies lower cognitive costs and is easier to operate than cognitive reappraisal (Troy et al., 2017).

Furthermore, psychometric measures alone cannot possibly capture a thorough and detailed explanation of the factors influencing performance in archery. Thus, the present results can be complemented by integrating skill constructs, fitness constructs and to shed light on the relative importance of archery performance factors and guide the training practice process. Besides, recent studies have also suggested that genetic polymorphisms are associated with athletes’ emotional control (Altamura et al., 2019) and sport performance (Ahmetov et al., 2016), and therefore, the inclusion of genetic polymorphism structure in the model could be considered and guide the athlete selection.

Finally, the differences in the experience levels and level of participation among the athletes could affect their psychological capability which may affect the result. This study only focused on an elite archery athlete population, and therefore, the applicability of the model is currently limited to this group. Future research could further explore the applicability of the model to archers with different levels of experience or participation, even to athletes in other sports that require movement of fine control and a high level of concentration, such as shooting and golf. Comparisons between athletes of different experience levels and between different sports will bring their own contribution to the field in practice and in the literature.

## Conclusion

The model constructed in this study has good reliability and validity, with three psychological constructs (sport-confidence, attention, and cognitive reappraisal) explaining 33.6% of the variance in archery performance. The total effects of the three mental constructs on archery performance were, in descending order, sport-confidence, attention, and cognitive reappraisal. Sport-confidence and attention are the priority components of archery mental training, in which sport-confidence development should include the development of state sport-confidence and trait sport-confidence, and attention training should also aim at attentional allocation, breadth, and shifting. In terms of emotion regulation, archery athletes should be guided to use cognitive reappraisal strategies when facing differentiated contexts, that is, cognitive reappraisal could regulate emotions and enhance sport-confidence, but its use in competition may potentially negatively affect performance by taking up cognitive resources and should directly improve sport-confidence or a develop a positive orientation to arouse excitement.

## Data Availability Statement

The raw data supporting the conclusions of this article will be made available by the authors, without undue reservation.

## Ethics Statement

Ethical review and approval was not required for the study on human participants in accordance with the local legislation and institutional requirements. Written informed consent to participate in this study was provided by the participants’ legal guardian/next of kin.

## Author Contributions

DW contributed to the experimental design, data collection and analysis, and drafting of the manuscript. TH contributed to the experimental design and revision of the manuscript. RL, QS, YW, XL, JQ, and LZ contributed to the data collection and examined the study. HY conceived of and examined the study and revised the manuscript. LC contributed to the experimental design and data collection and revised the manuscript. All authors contributed to the article and approved the submitted version.

## Funding

This work was supported by the Key Program of National Social Science Foundation (Grant No. 19ATY010).

## Conflict of Interest

The authors declare that the research was conducted in the absence of any commercial or financial relationships that could be construed as a potential conflict of interest.

## Publisher’s Note

All claims expressed in this article are solely those of the authors and do not necessarily represent those of their affiliated organizations, or those of the publisher, the editors and the reviewers. Any product that may be evaluated in this article, or claim that may be made by its manufacturer, is not guaranteed or endorsed by the publisher.

## References

[ref1] Abernethy B. Maxwell J. P. Masters R. S. W. Kamp J. V. D. Jackson R. C. (2007). “Attentional Processes in Skill Learning and Expert Performance,” in Handbook of Sport Psychology. eds. Tenenbaum G. Eklund R. C. (New York, NY: John Wiley & Sons), 245–263.

[ref2] Ahmetov I. Egorova E. Gabdrakhmanova L. Fedotovskaya O. (2016). Genes and athletic performance: an update. Med. Sport Sci. 61, 41–54. doi: 10.1159/000445240, PMID: 27287076

[ref3] Altamura M. Iuso S. D’Andrea G. D’Urso F. Piccininni C. Angelini E.. (2019). Maladaptive coping strategies and neuroticism mediate the relationship between 5HTT-LPR polymorphisms and symptoms of anxiety in elite athletes. Clin. Neuropsychiatry 16, 62–71. doi: 10.1101/493320, PMID: 34908940 PMC8650181

[ref4] Anmin L. Gang Q. Yang J. (1999). On the method of diagnosing and evaluating of Chinese elite athletes’ self confidence. J. Wuhan Inst. Phys. Educ. 33, 78–81. doi: 10.15930/j.cnki.wtxb.1999.03.025

[ref5] Arribas-Galarraga S. Saies E. Cecchini J. A. Arruza J. A. Luis De Cos M. I. (2017). The relationship between emotional intelligence, self-determined motivation and performance in canoeists. J. Hum. Sport Exerc. 12, 630–639. doi: 10.14198/jhse.2017.123.07

[ref6] Balk Y. A. Adriaanse M. A. de Ridder D. T. D. Evers C. (2013). Coping under pressure: employing emotion regulation strategies to enhance performance under pressure. J. Sport Exerc. Psychol. 35, 408–418. doi: 10.1123/jsep.35.4.408, PMID: 23966450

[ref7] Bandura A. (1977a). Self-efficacy: toward a unifying theory of behavioral change. Psychol. Rev. 84, 191–215. doi: 10.1037//0033-295x.84.2.191 847061

[ref8] Bandura A. (1997b). Self-efficacy: the exercise of control. J. Cogn. Psychother. 13:158

[ref9] Baumeister R. F. Bratslavsky E. Muraven M. Tice D. M. (1998). Ego depletion: is the active self a limited resource? J. Pers. Soc. Psychol. 74, 1252–1265. doi: 10.1037/0022-3514.74.5.1252, PMID: 9599441

[ref10] Bebetsos E. (2015). Psychological skills of elite archery athletes. J. Hum. Sport Exerc. 10, 623–628. doi: 10.14198/jhse.2015.102.0

[ref11] Brooks A. W. (2014). Get excited: reappraising pre-performance anxiety as excitement. J. Exp. Psychol. Gen. 143, 1144–1158. doi: 10.1037/a0035325, PMID: 24364682

[ref12] Buschman T. Miller E. (2010). Shifting the spotlight of attention: evidence for discrete computations in cognition. Front. Hum. Neurosci. 4:194. doi: 10.3389/fnhum.2010.00194, PMID: 21119775 PMC2990535

[ref13] Carson H. J. Collins D. (2016). The fourth dimension: a motoric perspective on the anxiety–performance relationship. Int. Rev. Sport Exerc. Psychol. 9, 1–21. doi: 10.1080/1750984X.2015.1072231, PMID: 26692896 PMC4662095

[ref14] Chin W. W. (1998). Issues and opinion on structural equation modeling. MIS Q. 22:1.

[ref15] Cisler J. M. Olatunji B. O. Feldner M. T. Forsyth J. P. (2010). Emotion regulation and the anxiety disorders: an integrative review. J. Psychopathol. Behav. Assess. 32, 68–82. doi: 10.1007/s10862-009-9161-1, PMID: 20622981 PMC2901125

[ref002] Cohen J. (1988). “CHAPTER 9 - Multiple Regression and Correlation Analysis,” in Statistical Power Analysis for the Behavioral Sciences. ed. Cohen J. (New York, NY: Routledge), 407–450.

[ref16] Desbordes G. Gard T. Hoge E. A. Hölzel B. K. Kerr C. Lazar S. W.. (2015). Moving beyond mindfulness: defining equanimity as an outcome measure in meditation and contemplative research. Mindfulness 6, 356–372. doi: 10.1007/s12671-013-0269-8 PMC4350240 25750687

[ref17] Efinger L. Thuillard S. Dan-Glauser E. S. (2019). Distraction and reappraisal efficiency on immediate negative emotional responses: role of trait anxiety. Anxiety Stress Coping 32, 412–427. doi: 10.1080/10615806.2019.1597859, PMID: 31001990

[ref18] Eysenck M. W. Calvo M. G. (1992). Anxiety and performance: the processing efficiency theory. Cognit. Emot. 6, 409–434. doi: 10.1080/02699939208409696, PMID: 35310248

[ref19] Eysenck M. W. Derakshan N. (2011). New perspectives in attentional control theory. Personal. Individ. Differ. 50, 955–960. doi: 10.1016/j.paid.2010.08.019, PMID: 22364371

[ref20] Eysenck M. W. Nazanin D. Rita S. Calvo M. G. (2007). Anxiety and cognitive performance: attentional control theory. Emotion 7, 336–353. doi: 10.1037/1528-3542.7.2.336, PMID: 17516812

[ref21] Feldman G. Lavallee J. Gildawie K. Greeson J. M. (2016). Dispositional mindfulness uncouples physiological and emotional reactivity to a laboratory stressor and emotional reactivity to executive functioning lapses in daily life. Mindfulness 7, 527–541. doi: 10.1007/s12671-015-0487-3, PMID: 27087863 PMC4831864

[ref22] Fornell C. Larcker D. F. (1981). Evaluating structural equation models with unobservable variables and measurement error. J. Mark. Res. 18, 39–50. doi: 10.1177/002224378101800104

[ref23] Fung L. Ng J. K. Cheung S. Y. (2001). Confirmatory factor analysis of the trait sport-confidence inventory and state sport-confidence inventory on a Chinese sample. Int. J. Sport Psychol. 32, 304–313.

[ref24] Gan S. Yang J. Chen X. Zhang X. Yang Y. (2017). High working memory load impairs the effect of cognitive reappraisal on emotional response: evidence from an event-related potential study. Neurosci. Lett. 639, 126–131. doi: 10.1016/j.neulet.2016.12.069, PMID: 28041962

[ref25] General Administration of Sport of China (2019). *Technical Hierarchy of Archery Athlete*. General Administration of Sport of China. Available at: https://www.sport.gov.cn/n4/n207/n209/n23554520/c23616305/content.html (Accessed April 1, 2022).

[ref26] Gonzalez C. C. Causer J. Grey M. J. Humphreys G. W. Miall R. C. Williams A. M. (2017). Exploring the quiet eye in archery using field- and laboratory-based tasks. Exp. Brain Res. 235, 2843–2855. doi: 10.1007/s00221-017-4988-2, PMID: 28660285 PMC5550539

[ref27] Gross J. J. (2010). Emotion regulation: affective, cognitive, and social consequences. Psychophysiology 39, 281–291. doi: 10.1017/S0048577201393198 12212647

[ref28] Gross J. J. John O. P. (2003). Individual differences in two emotion regulation processes: implications for affect, relationships, and well-being. J. Pers. Soc. Psychol. 85, 348–362. doi: 10.1037/0022-3514.85.2.348, PMID: 12916575

[ref29] Gross J. J. Thompson R. A. (2007). “Emotion Regulation: Conceptual Foundations,” in Handbook of emotion regulation. ed. Gross J. J. (New York, NY: The Guilford Press), 3–24.

[ref30] Hair J. F. Howard M. C. Nitzl C. (2020). Assessing measurement model quality in PLS-SEM using confirmatory composite analysis. J. Bus. Res. 109, 101–110. doi: 10.1016/j.jbusres.2019.11.069

[ref31] Hair J. F. Hult G. T. M. Ringle C. M. Sarstedt M. (2017). A Primer on Partial Least Squares Structural Equation Modeling (PLS-SEM). 2nd Edn. Los Angeles, CA: Sage Publications.

[ref32] Hair J. F. Ringle C. M. Sarstedt M. (2011). PLS-SEM: indeed a silver bullet. J. Mark. Theory Pract. 19, 139–152. doi: 10.2753/MTP1069-6679190202

[ref33] Hair J. F. Sarstedt M. Ringle C. M. Mena J. A. (2012). An assessment of the use of partial least squares structural equation modeling in marketing research. J. Acad. Mark. Sci. 40, 414–433. doi: 10.1007/s11747-011-0261-6

[ref34] Hays K. Thomas O. Maynard I. Bawden M. (2009). The role of confidence in world-class sport performance. J. Sports Sci. 27, 1185–1199. doi: 10.1080/02640410903089798, PMID: 19724964

[ref35] Henseler J. Ringle C. M. Sarstedt M. (2015). A new criterion for assessing discriminant validity in variance-based structural equation modeling. J. Acad. Mark. Sci. 43, 115–135. doi: 10.1007/s11747-014-0403-8

[ref36] Hofmann S. G. Heering S. Sawyer A. T. Asnaani A. (2009). How to handle anxiety: The effects of reappraisal, acceptance, and suppression strategies on anxious arousal. Behav. Res. Ther. 47, 389–394. doi: 10.1016/j.brat.2009.02.010, PMID: 19281966 PMC2674518

[ref37] Indahwati N. Ristanto K. (2016). The application of pettlep imagery exercise to competitive anxiety and concentration in Surabaya archery athletes. Int. J. Educ. Sci. Res. 6, 131–138.

[ref38] Jamieson J. P. Mendes W. B. Nock M. K. (2013). Improving acute stress responses: the power of reappraisal. Curr. Dir. Psychol. Sci. 22, 51–56. doi: 10.1177/0963721412461500

[ref39] Kaur S. Shenoy S. (2019). A study on the relationship of trait and state anxiety on the performance of archers. Eur. J. Physic. Educ. Sport Sci. 5, 95–105.

[ref40] Keng S. Robins C. J. Smoski M. J. Dagenbach J. Leary M. R. (2013). Reappraisal and mindfulness: a comparison of subjective effects and cognitive costs. Behav. Res. Ther. 51, 899–904. doi: 10.1016/j.brat.2013.10.006, PMID: 24225174 PMC4030543

[ref41] Kim H. Kim S. So W. (2015). The relative importance of performance factors in korean archery. J. Strength Cond. Res. 29, 1211–1219. doi: 10.1519/JSC.0000000000000687, PMID: 25226316

[ref42] Kim D. Oh K. (2015). The relationship between emotion and emotion regulation and performance in archery athletes. Korean Soc. Sport Psychol. 26, 31–46. doi: 10.14385/KSSP.26.4.31ISSN1226-685X

[ref43] Kubiak J. Rother S. Egloff B. (2019). Keep your cool and win the game: emotion regulation and performance in table tennis. J. Pers. 87, 996–1008. doi: 10.1111/jopy.12451, PMID: 30638260

[ref44] Kucharski B. Strating M. A. Ahluwalia Cameron A. Pascual-Leone A. (2018). Complexity of emotion regulation strategies in changing contexts: a study of varsity athletes. J. Contextual Behav. Sci. 10, 85–91. doi: 10.1016/j.jcbs.2018.09.002

[ref45] Lane A. M. Devonport T. J. Friesen A. P. Beedie C. J. Fullerton C. L. Stanley D. M. (2016). How should I regulate my emotions if I want to run faster? Eur. J. Sport Sci. 16, 465–472. doi: 10.1080/17461391.2015.1080305, PMID: 26361078

[ref46] Lee K. (2009). Evaluation of attention and relaxation levels of archers in shooting process using brain wave signal analysis algorithms. Korean J. Sci. Emot. Sensib. 12, 341–350.

[ref47] Lim I. S. (2016). Correlation between salivary alpha-amylase, anxiety, and game records in the archery competition. J. Exerc. Nutr. Biochem. 20, 44–47. doi: 10.20463/jenb.2016.0050, PMID: 28150473 PMC5545204

[ref48] Love S. Kannis-Dymand L. Lovell G. P. (2018). Metacognitions in triathletes: associations with attention, state anxiety, and relative performance. J. Appl. Sport Psychol. 30, 421–436. doi: 10.1080/10413200.2018.1440660

[ref001] Lu Q. Li P. Wu Q. Liu X. Wu Y. (2021). Efficiency and enhancement in attention networks of elite shooting and archery athletes. Front. Psychol. 12:527. doi: 10.3389/fpsyg.2021.638822 PMC7985157 33767650

[ref49] Marcoulides G. A. Saunders C. (2006). Editor’s comments: PLS: a silver bullet? MIS Q. 30, iii–ix. doi: 10.2307/25148727

[ref50] Martens R. Burton D. Vealey R. S. Bump L. A. Smith D. E. (1990). “The development of the competitive state anxiety Inventory-2 (CSAI-2),” in Competitive Anxiety in Sport. eds. Martens R. Vealey R. S. Burton D. (Champaign, IL: Human Kinetics), 117–190.

[ref51] Martin J. J. Gill D. L. (1991). The relationships among competitive orientation, sport-confidence, self-efficacy, anxiety, and performance. J. Sport Exerc. Psychol. 13, 149–159. doi: 10.1123/jsep.13.2.149

[ref52] McRae K. Jacobs S. E. Ray R. D. John O. P. Gross J. J. (2012). Individual differences in reappraisal ability: links to reappraisal frequency, well-being, and cognitive control. J. Res. Pers. 46, 2–7. doi: 10.1016/j.jrp.2011.10.003

[ref53] Mellalieu S. D. Hanton S. Fletcher D. (2009). A Competitive Anxiety Review: Recent Directions in Sport Psychology Research. New York, NY: Nova Science Publishers Inc.

[ref54] Mellalieu S. D. Hanton S. O’Brien M. (2004). Intensity and direction of competitive anxiety as a function of sport type and experience. Scand. J. Med. Sci. Sports 14, 326–334. doi: 10.1111/j.1600-0838.2004.00389.x, PMID: 15387807

[ref55] Moran A. P. (1996). The psychology of concentration in sport performers: a cognitive analysis. Int. J. Sport Psychol. 36:86.

[ref56] Moran A. (2012). Sport and Exercise Psychology: A Critical Introduction. 2nd Edn. London: Routledge.

[ref57] Mun C. (2011). The influences of self-management and psychological skills on concentration among elite shooting athletes. J. Coach. Dev. 13, 47–57.

[ref58] Ochsner K. N. Gross J. J. (2005). The cognitive control of emotion. Trends Cogn. Sci. 9, 242–249. doi: 10.1016/j.tics.2005.03.010, PMID: 15866151

[ref59] Ortner C. N. M. Marie M. S. Corno D. (2016). Cognitive costs of reappraisal depend on both emotional stimulus intensity and individual differences in habitual reappraisal. PLoS One 11:e167253. doi: 10.1371/journal.pone.0167253, PMID: 27936022 PMC5147884

[ref60] Pei G. Song-ping Y. (2007). Contradiction on traditional collimation method in archery. J. Beijing Sport Univ. 30, 564–566. doi: 10.3969/j.issn.1007-3612.2007.04.045

[ref61] Peterson R. A. Kim Y. (2013). On the relationship between coefficient alpha and composite reliability. J. Appl. Psychol. 98, 194–198. doi: 10.1037/a0030767, PMID: 23127213

[ref62] Richards J. M. Gross J. J. (2000). Emotion regulation and memory: the cognitive costs of keeping one’s cool. J. Pers. Soc. Psychol. 79, 410–424. doi: 10.1037/0022-3514.79.3.410, PMID: 10981843

[ref63] Ringle C. M. Wende S. Becker J. (2015). SmartPLS 3. Boenningstedt: SmartPLS GmbH.

[ref64] Rosenqvist O. Skans O. N. (2015). Confidence enhanced performance?–The causal effects of success on future performance in professional golf tournaments. J. Econ. Behav. Organ. 117, 281–295. doi: 10.1016/j.jebo.2015.06.020

[ref65] Salleh F. N. M. Hashim H. A. Krasilshchikov O. (2020). Determination of psychological correlates of peak performance in developmental archers. J. Phys. Educ. Sport 20, 344–347. doi: 10.7752/jpes.2020.s1048

[ref66] Sammy N. Anstiss P. A. Moore L. J. Freeman P. Wilson M. R. Vine S. J. (2017). The effects of arousal reappraisal on stress responses, performance and attention. Anxiety Stress Coping 30, 619–629. doi: 10.1080/10615806.2017.1330952, PMID: 28535726

[ref67] Sarro K. J. Viana T. D. C. De Barros R. M. L. (2021). Relationship between bow stability and postural control in recurve archery. Eur. J. Sport Sci. 21, 515–520. doi: 10.1080/17461391.2020.1754471, PMID: 32267203

[ref68] Sarstedt M. Ringle C. M. Hair J. F. (2017). “Partial least squares structural equation modeling,” in Handbook of Market Research. eds. Homburg C. Klarmann M. Vomberg A. (Cham, Switzerland: Springer International Publishing), 1–40.

[ref69] Shen L. K. (2004). An empirical test of the structural model of Chinese athletes’ sports self-confidence. master. Beijing Sport University: Beijing.

[ref70] Sheppes G. Meiran N. (2007). Better late than never? On the dynamics of online regulation of sadness using distraction and cognitive reappraisal. Personal. Soc. Psychol. Bull. 33, 1518–1532. doi: 10.1177/0146167207305537, PMID: 17933748

[ref71] Sheppes G. Meiran N. (2008). Divergent cognitive costs for online forms of reappraisal and distraction. Emotion 8, 870–874. doi: 10.1037/a0013711, PMID: 19102598

[ref72] Soylu A. R. Ertan H. Korkusuz F. (2006). Archery performance level and repeatability of event-related EMG. Hum. Mov. Sci. 25, 767–774. doi: 10.1016/j.humov.2006.05.002, PMID: 16859789

[ref73] Stanger N. Chettle R. Whittle J. Poolton J. (2018). The role of preperformance and in-game emotions in cognitive interference during sport performance: the moderating role of self-confidence and reappraisal. Sport Psychol. 32, 114–124. doi: 10.1123/tsp.2017-0001

[ref74] Stuart J. Atha J. (1990). Postural consistency in skilled archers. J. Sports Sci. 8, 223–234. doi: 10.1080/02640419008732148, PMID: 2084269

[ref75] Sun Y. Zhang L. Xiaodong L. I. Jian L. I. Xiufeng W. U. Section S. P.. (2013). Role of ego depletion in suboptimal performance: evidence from qualitative and experimental research. J. Tianjin Univ. Sport 28, 277–286. doi: 10.13297/j.cnki.issn1005-0000.2013.04.006

[ref76] Taha Z. Musa R. Abdullah M. Maliki A. B. Kosni N. Mat-Rasid S. M.. (2018). Supervised pattern recognition of archers’ relative psychological coping skills as a component for a better archery performance. J. Fundam. Appl. Sci. 10, 10–467. doi: 10.4314/jfas.v10i1s.33

[ref77] Taherdoost H. (2016). Validity and reliability of the research instrument; how to test the validation of a questionnaire/survey in a research. Int. J. Acad. Res. Manag. 5, 28–36. doi: 10.2139/ssrn.3205040

[ref78] Tomé-Lourido D. Arce Fernández C. Ponte Fernández D. (2019). The relationship between competitive state anxiety, self-confidence and attentional control in atletes. Rev. Psicol. Deport. 28, 143–150.

[ref79] Troy A. S. Shallcross A. J. Brunner A. Friedman R. Jones M. C. (2017). Cognitive reappraisal and acceptance: effects on emotion, physiology, and perceived cognitive costs. Emotion 18, 58–74. doi: 10.1037/emo0000371, PMID: 29154585 PMC6188704

[ref80] Uphill M. Lane A. Jones M. (2012). Emotion regulation questionnaire for use with athletes. Psychol. Sport Exerc. 13, 761–770. doi: 10.1016/j.psychsport.2012.05.001, PMID: 35270288

[ref81] Urbach N. Ahlemann F. (2010). Structural equation modeling in information systems research using partial least squares. J. Inf. Technol. Theory Appl. 11, 5–40.

[ref82] Vealey R. S. (1986). Conceptualization of sport-confidence and competitive orientation: preliminary investigation and instrument development. J. Sport Psychol. 8, 221–246. doi: 10.1123/jsp.8.3.221

[ref83] Wagstaff C. R. D. (2014). Emotion regulation and sport performance. J. Sport Exerc. Psychol. 36, 401–412. doi: 10.1123/jsep.2013-0257, PMID: 25226609

[ref84] Wang S. B. Liu D. Q. (2013). Theory research on the winning factors in archery tournament. J. Beijing Sport Univ. 36, 129–134. doi: 10.19582/j.cnki.11-3785/g8.2013.09.024

[ref85] Wang L. Liu H. Li Z. (2007). Reliability and validity of emotion regulation questionnaire Chinese revised version. Chin. J. Health Psychol. 15, 503–505. doi: 10.13342/j.cnki.cjhp.2007.06.012

[ref86] Weinberg R. S. Gould D. (2014). Foundations of Sport and Exercise Psychology. Champaign, IL: Human Kinetics.

[ref87] Woodman T. Hardy L. (2003). The relative impact of cognitive anxiety and self-confidence upon sport performance: a meta-analysis [journal article; meta-analysis]. J. Sports Sci. 21, 443–457. doi: 10.1080/0264041031000101809, PMID: 12846532

[ref88] Yang H. Ting W. Shi Y. (2008). Research on talent identification indexes and criterions of excellent archers in China. China Sport Sci. 28, 33–43. doi: 10.16469/j.css.2008.05.007

[ref89] Yin H. C. Zhang F. Z. Song X. Q. (2006). Research of measurement and evaluation on attention of elite athletes. China Sport Sci. 03, 58–63. doi: 10.16469/j.css.2006.03.012

[ref90] Zhao G. Wei L. Zhao X. Li K. (2013). The intervention effect of competition scheme and pre-performance routines to the performance strategies, pre-competition mental state and performance of the elite archers. China Sport Sci. 33, 39–48. doi: 10.16469/j.css.2013.12.012

